# Opening up ideas: an advent calendar for patient and public engagement in clinical trials research

**DOI:** 10.1186/s40900-023-00530-6

**Published:** 2023-12-11

**Authors:** Nicola L. Harman, Kerrie McGiveron, Catrin Tudur Smith, Paula R. Williamson, Heather Barrington

**Affiliations:** Health Data Science, Institute of Population Health, University of Liverpool, Block F Waterhouse Building, Liverpool, UK

**Keywords:** Patient and public involvement, Patient and public engagement, Clinical trials, Clinical trials methodology

## Abstract

The involvement of patients and the public in research is now an expectation in research with funders requesting a clear plan of involvement and engagement. In the United Kingdom involvement typically focuses on research prioritisation, design and delivery, in contrast activities that share the results of research or research methods more generally are considered to be engagement. Clinical trials tend to concentrate on involvement activities with less emphasis on engagement. To promote engagement activities in the context of clinical trials we asked people attending the 2022 International Clinical Trials Methodology Conference to share ideas on how we can best engage with patients and the public. Responses were reviewed and 22 themes identified. One suggestion was to create an advent calendar and so these 22 themes plus two from the authors were used as a daily tweet from the 1st to the 24th December 2022. Here we share these ideas and draw comparisons between engagement activities in research and traditions of the Christmas period. The ideas shared are not intended as a definitive list but instead a novel way to start discussions between experts by experience, researchers, health professionals and communities to facilitate co-production of meaningful engagement strategies.

**Supplementary Information:**

The online version contains supplementary material available at 10.1186/s40900-023-00530-6.

## Abstract (plain language summary)

Patient and public involvement and engagement are terms used to describe specific activities that have a variety of goals from information giving through topublic co-production of research. Involvement and engagement are important as they can help reduce waste in research by ensuring that the research is relevant, conducted well and that the results are shared to those that will use them to make decisions about treatments, including patients. In the United Kingdom the term “engagement” usually refers to activities that focus mainly on information giving, for example sharing the results of research or information about how research is done in general. In this commentary we share ideas for engagement activities that were collected from people attending the International Clinical Trials Methodology Conference in 2022. One of the ideas shared was to have an advent calendar and we have used this to draw comparisons between traditions surrounding the Christmas period and engagement of patients and the public. We share 24 different ideas in the form of a printable advent calendar and invite the clinical trials community, including experts by experience, to reflect on these to generate more ideas for meaningful engagement activities so that everyone who will use the results of research has the opportunity to shape, share, and benefit from research.

**Supplementary Information:**

The online version contains supplementary material available at 10.1186/s40900-023-00530-6.

## Background

For many people across the world, and in the UK, the festive season in December is a time of interaction and engagement, where we actively consult others on their hopes and wishes, collaborate with our nearest and dearest to seek out perfect presents, and aim to inform and inspire our family, friends, and most importantly Father Christmas, so that we aren’t disappointed on Christmas morning.

These activities are akin to some of the engagement activities described in the layers in the Wellcome Trust engagement onion [1] and modified University College Dublin public engagement spectrum avocado [2]. Although you might only expect to see this food, the onion not the avocado, mixed with sage and breadcrumbs and stuffed in a turkey or nutloaf, we can, with the spirit of Christmas, seek inspiration for involvement and engagement beyond the dinner table and into clinical trials research.

Patient and public involvement and engagement are terms used to describe a range of activities in research. In the context of clinical trials research in the United Kingdom, involvement typically describes research that is conducted “with” or “by” patients or members of the public [3]. This involvement includes collaborative partnerships and shared-decision making between members of the public, with relevant knowledge or experience, and researchers. Involvement can be in any or all elements of a clinical trial including research question prioritisation, design, delivery, oversight, analysis, and dissemination.

The term “engagement” is often used interchangeably with “involvement”, and is the preferred term in some countries, making it challenging to unpick the different activities within the context of clinical trials. Although a contested term globally, in the United Kingdom, engagement refers to interactions with a defined audience to share information or start a dialogue about a particular project or research area [4]. As one might expect there is overlap between what is considered involvement and engagement, particularly with activities that focus on shared dialogue rather than information giving. Indeed, they are complementary elements where good engagement can lead to a desire for involvement and spark new research ideas, and involvement can generate ideas for wider engagement. In this paper we take guidance from the National Institute of Health research (NIHR) and use the term “engagement” to refer to activities that focus on the provision and dissemination of knowledge, and “involvement” for research activities that are carried out with or by members of the public [4].

Involvement of patients and the public in research brings expertise and knowledge that has the potential to reduce research waste [5] by ensuring that research is relevant and that the study is designed, delivered and disseminated in a patient centred way [6]. The benefits of involving patients and the public in research, for example, positive effects on recruitment [7], are recognised by research funders, with many funders promoting involvement by requiring a detailed, and appropriately costed involvement strategy [8]. Much of this focuses on activities related to research prioritisation, trial design and delivery [9], perhaps because only around half of trials achieve their recruitment target [10].

Although less emphasis has been given to activities co-designed with the public to share the results of the research [9], engagement and dissemination activities have the potential to positively influence recruitment and retention not only through involvement in the delivery of a trial [7] but also by promoting general awareness and knowledge [11, 12]. In a survey of 12,427 people, the majority of respondents had not previously taken part in a research study (n = 10,233). Of these 10,233, 25% of participants self-reported a very good understanding of the term “clinical” trial. For the 2,194 who had taken part in a trial before, 58% in self-reported a very good understanding. But, when probed further all those reporting good/very good understanding, irrespective of prior participation, had a somewhat superficial knowledge with respect to where clinical trials were conducted and agencies responsible for oversight of safety [13]. Nevertheless, knowledge gained from previous participation may have influenced willingness to take part in trials, 59% of participants with previous trial participation were very willing to take part in a clinical trial compared to just 25% of those with no prior experience [13].

Transparent, engaging communication that makes research accessible to the public and communities is important as many, if not all, will access health care, and benefit from the evidence generated by trials at some point in their lifetime. It is important that the clinical trials research community, including patient and public research partners, reflect on whether we are engaging the public at all layers of the engagement onion and if not, how we can work collaboratively to identify gaps in engagement and develop strategies to fill these. This is particularly important for trials methodology or ‘research on research’ where we want to facilitate understanding of why we need to test and evaluate the ways we design and run clinical trials, and not just the interventions themselves [14].

### Generating ideas for engagement

It is at this point that we can look outside of clinical trials to other areas with public engagement models. For example, the North Pole Organisation for Present Delivery, led by Father Christmas, undertakes public engagement with large reach and impact. The engagement strategy of Father Christmas and team encompasses a range of activities, for a specific target population, that capture attention and create excitement. And whilst Father Christmas is the figurehead of these activities he extends his reach via a team of elves and reindeer. Engagement activities range from in person pop-up events, through to mass mailings and pop songs. Together these create a dialogue between the general public and the North Pole that set priorities and have clear lines of communication…preferably letters by post [15].

**Fig. 1:**
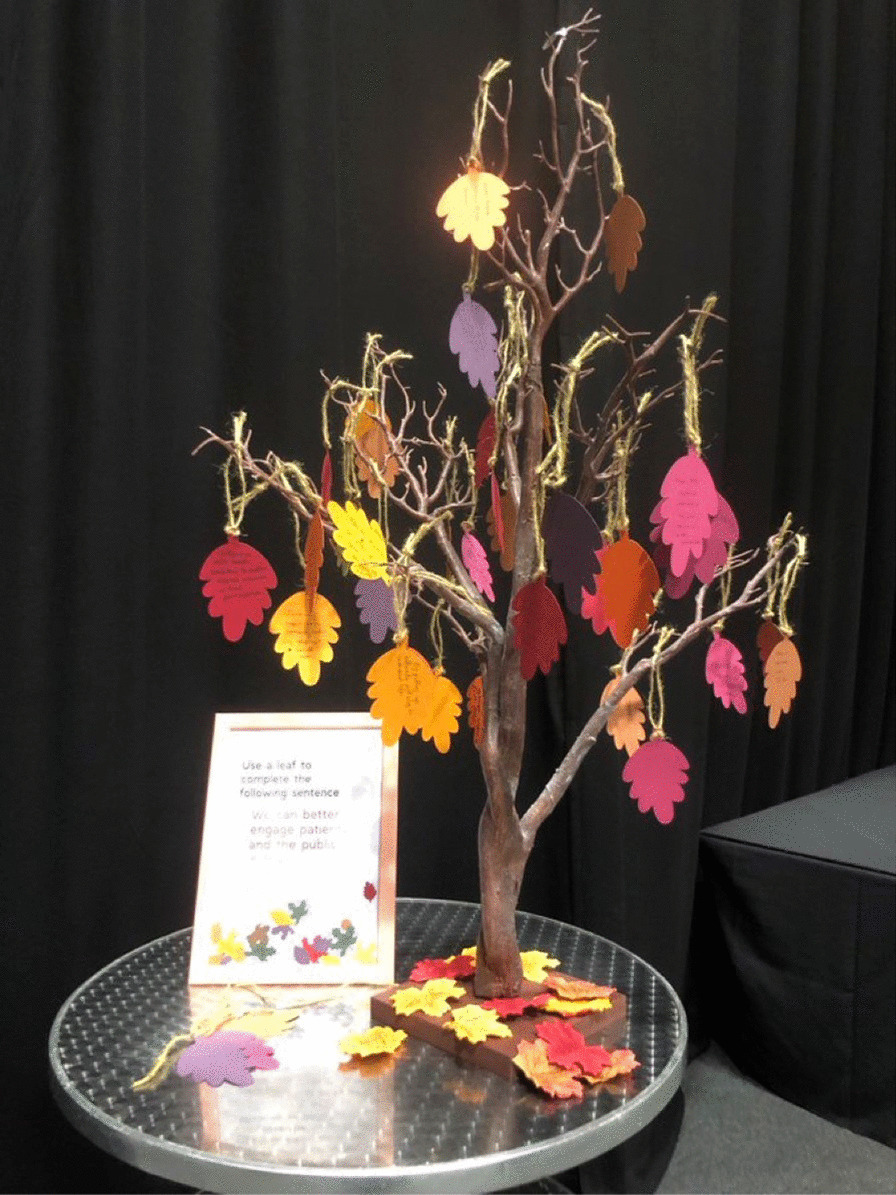
The ideas tree in situ at the International Clinical Trials Methodology Conference in Harrogate, October 2022

But can we generate better engagement ideas than the North Pole and what might engagement activities look like for clinical trials research? To find out we made a list and checked it twice. More specifically we invited delegates of the 2022 International Clinical Trials Methodology Conference (ICTMC 2022), including patient research partners, to anonymously complete the question “We can best engage patients and the public with trials methodology by …". Responses were written on a paper leaf that was added to a tree. Leaves were custom made using a Silhouette America © Portrait cutting machine. This tree, leaves and question were used as an activity in the Trials Methodology Research Partnership, Doctoral Training Partnership (TMRP DTP) student session and then displayed in the central refreshment and poster viewing area, open to all delegates, for additional responses (Fig. 1). There were no restrictions on the number or type of ideas an individual could submit. The question was displayed in a frame next to the tree and blank, pre-strung, leaves and pens provided.

At the end of the conference, leaves were collected and responses transcribed verbatim, in Microsoft Excel, to create our initial list. This list was then checked twice by two authors (NLH and HB), ideas submitted were grouped where appropriate and themes identified.

Fifty-nine responses were received over the course of the three days, including those submitted as part of a specific session for the TMRP DTP students. Three responses did not provide enough information to be reviewed.

**Fig. 2:**
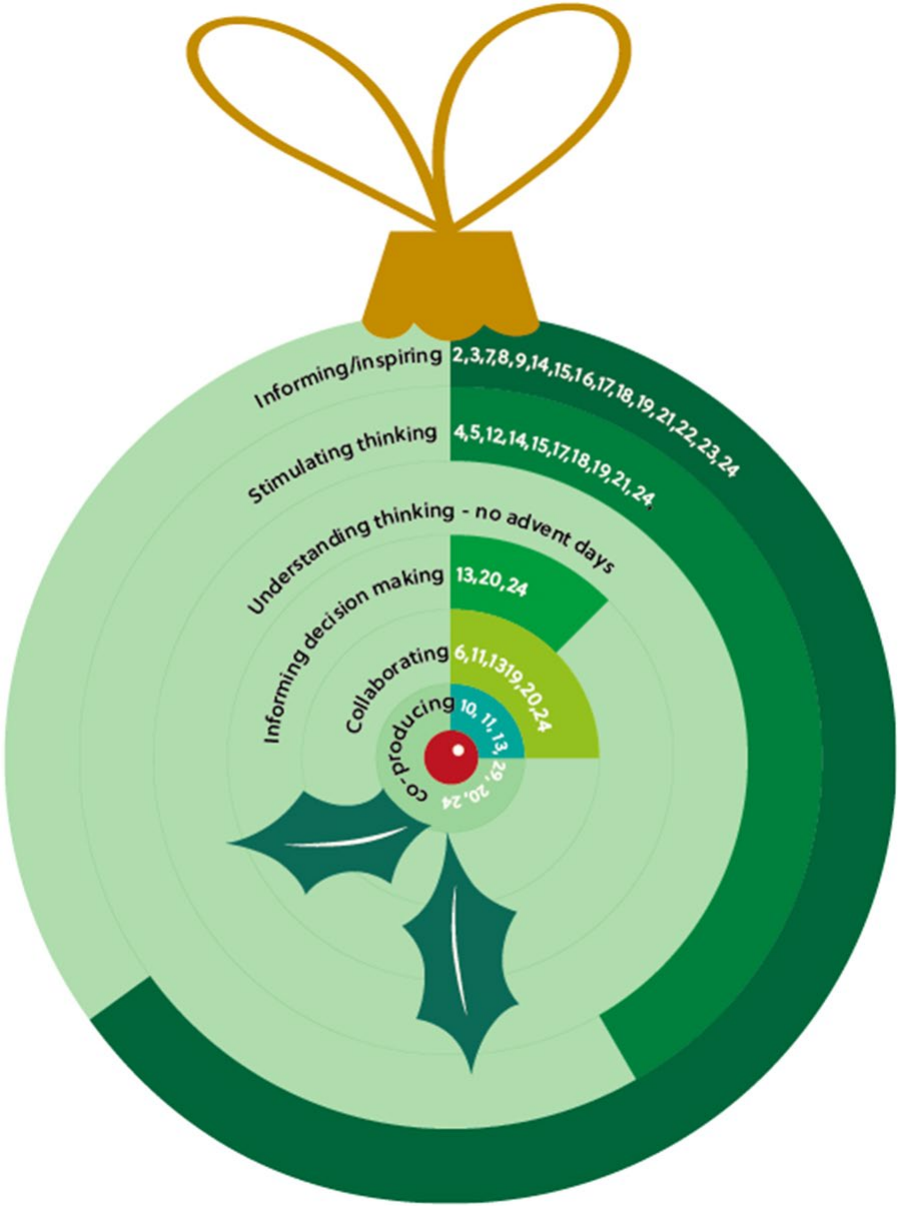
Overview of the days of the advent calendar and which of the six broad types of engagement activity they were categorised as

Twenty-two themes were identified that represented ideas for engaging the public with clinical trials research. Responses were compared to six overarching activity types [2] (Fig. 2). The majority of ideas were categorised as “informing/inspiring” and “stimulating thinking”. Fewer themes were identified in the “informing decision making”, “collaboration” and “co-production” categories, reflecting that, in clinical trials research, these activities are usually termed patient and public involvement rather than engagement and perhaps considered outside of the scope of the question asked at the conference. No responses were categorised as “understanding thinking” activities, which include surveys, opinion polls and consultations [2]. This may be because in the trials community these were considered as specific research studies or activities and not engagement.

There was also a specific suggestion of an advent calendar of ideas for researchers and this was used as a novel way to share the themes identified and seek further input from the trials community. The advent calendar was delivered using Twitter (now X) in December 2022 (1st–24th) with one theme shared as a daily tweet that noted relevant initiatives and invited additional opinions. Each tweet included an animated GIF created using Procreate® for iPad. As there were only 22 themes, an additional item for day 23 was suggested by authors NLH and HB, and the 24th day was a thank you from the authors and an acknowledgement that as a community we need to work together to action some of the suggestions. Engagement with each tweet varied, the average impressions across the 24 days was 1512 (range 711–7143). The highest engagement, including likes, impressions, and shares, was observed on day 1 (7143 impressions), for the tweet “Involving public contributors from the very start”. Tweets on days 2-24 averaged 1267 impressions (range 711–1911) with no particular impact of the day of the week that the post was shared. Engagement with tweets was predominantly via viewing, liking or re-tweeting with less interaction from comments. Full details of each tweet including the content and types of engagement are available in Additional file 1.

## Discussion

### An advent calendar as a way of sharing information

The idea collection activity and advent calendar were a novel and fun way to brainstorm and share ideas relevant to the engagement of the public with clinical trials. The approach we describe, including anonymous responses, meant that there were no restrictions on ideas allowing brainstorming irrespective of cost or practicality. In our example, participation in idea generation was restricted to those attending the ICTMC 2022 conference and the views represented are specific to this group. But, this method could be applied in other settings both in person or digitally to incorporate a broader range of perspectives. In particular consideration should be given to how this or other creative methods could be used to collaborate and engage with different groups of people, including under-served groups [16], to support idea generation and the design and delivery of engagement activities.

**Fig. 3:**
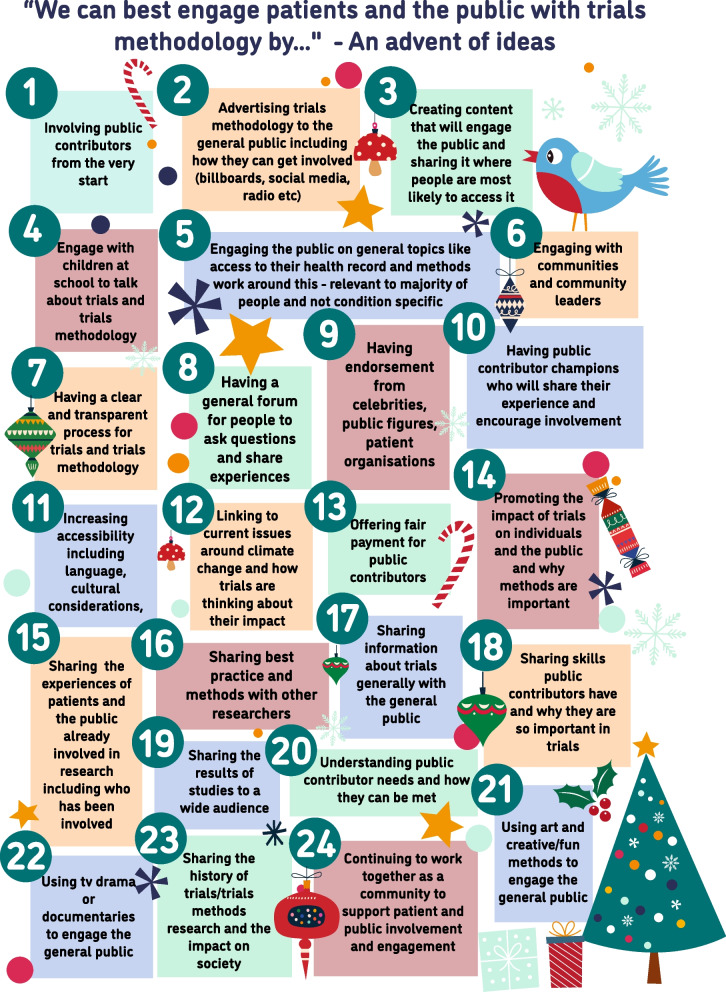
An advent calendar of all 24 themes shared

In this report we share ideas for involvement as an advent calendar. All ideas can be opened up in Fig. 3, printable files and instructions to make your own advent calendar are available online [17]. We have used a specific holiday as an example of a fun way to share information and to create interest in engagement activities for trials and trials methodology. Using an advent calendar has the benefit of being something familiar with a format well suited to breaking down information into smaller, easier to understand parts. The format also creates anticipation for what comes next. Despite these benefits, this approach is limited by being suitable only for a specific audience that celebrates Christmas. Whilst a broad appeal is perhaps desirable, and indeed many of the individual suggestions specifically referred to the “general public” as the target audience, it is important to recognise that the “general public” is not a homogenous group. Instead engagement activities benefit from a clearly defined and specific audience and from co-production with the populations and communities that they aim to engage. To fully meet the UK standards of involvement inclusivity standard for communications [18] further work is needed to consider how to appeal to different communities and information needs.

### Reflection and evaluation

Asking a simple question and providing a low-tech way to respond anonymously yielded a range of ideas and opportunities that could be taken forward in future work. Because of the minimal equipment needed this activity could easily be run in other locations including schools, cafes, libraries etc. with modification of the starting question to best reflect the knowledge and experience of those who might take part.

Although this activity was focused on “engagement” rather than “involvement” there is important learning from the UK standards of involvement [18], in particular the standards for “working together”, “inclusive opportunities”, “communications” and “impact”. For example, there is scope to increase the accessibility of the activity, and provide a more inclusive opportunity to be involved by sharing the activity more widely, by allowing people to share or record voice notes or to host the activity online. The sharing of ideas anonymously and for these to be considered irrespective of role or experience values all contributions but there could be further work to co-design the starting question with the public. Our approach to communication has been to use plain language and to write in an engaging and accessible way for all outputs. But, when, where and how engagement activities are shared is important, and benefits from discussion with target groups to understand how best to mobilise knowledge and opportunities.

The impact of this engagement activity has yet to be reviewed. To understand whether or not the idea generation activity and dissemination as an advent calendar was useful we need to know: whether any of the ideas have been taken forward; if they have, what has been done; and if not, what are the perceived barriers or facilitators for doing so e.g. relevance, cost, training etc. Engagement activities may go underreported in trials and trials methodology research and this impacts on the availability of shared knowledge. By sharing the activity and  the advent calendar we aim to promote discussion and dissemination of engagement strategies and in doing so maximise the potential to assess impact and improve how we engage with the public in the future.

### Existing and future work to facilitate engagement with trials and trials methodology

Some of the ideas shared link to existing projects. For example, the Schools Teaching Awareness of Randomised Trials (START) [19], The People’s Trial [20] and The Kid’s Trial [21] initiatives are examples of citizen science and engagement, that go beyond information giving, with schools (advent day 4) and the public (advent day 17). There are also examples of existing projects that aim to share information about trials methodology (day 2) [22, 23], that use creative and participatory methods to explore and share experiences of health care and health care research (advent day 21) [24, 25], and that provide guidance and support relevant to fair payment (day 13) [26] and wider involvement (day 6 and 11) [16, 27]. These examples are not exhaustive and neither are the ideas shared. Future work might include adding to this through engagement with other groups of people, the prioritisation of activities, or the development of guidance and training to support researchers and patient research partners to create and deliver engagement activities. Ideally these engagement activities would be co-produced with the public, with appropriate funding for public partners.

## Conclusions

Engagement activities may be undertaken less frequently in trials due to a focus on trial delivery/conduct. However, there are benefits to disseminating information about trials and trials methodology more widely. As a community, of trialists, methodologists and patient and public research partners, we have an opportunity to work together by using existing networks [28] or establishing new ones to develop creative and meaningful engagement and involvement activities, so that all those who use the results of the research have the opportunity to shape, share, and benefit from that research.

### Supplementary Information

**Additional file 1**. Full details of each tweet shared on the X platform on the 1st-24th December 2022. Engagement analytics are those provided by the X platform.

## Data Availability

All data generated or analysed during this study are included in this published article [and its supplementary information files].
