# Supplementary material for: Opening up ideas: an advent calendar for patient and public engagement in clinical trials research
Source: Res Involv Engagem. 2023 Dec 11;9:118. doi: 10.1186/s40900-023-00530-6 (PMC10714500; doi:10.1186/s40900-023-00530-6)
Supplement: Supplementary file 1 — Additional file 1. Full details of each tweet shared on the X platform on the 1st-24th December 2022. Engagement analytics are those provided by the X platform. [file 40900_2023_530_MOESM1_ESM.docx]

| **Supplementary file 1. – All tweets were shared by HB, @hetbag, on twitter.com** | | | | | | | | | | | | |
| --- | --- | --- | --- | --- | --- | --- | --- | --- | --- | --- | --- | --- |
| **Day** | **Text content of animated GIF image** | **Domain** | **Tweet** | **Retweets (including re-tweets of quoted tweets)** | **Quote tweets** | **Total Likes including likes of quoted tweets** | **Bookmarks** | **Twitter impressions**  **(31-07-23)** | **Twitter Engagements**  **(31-07-23)** | **Comments** | **Day of the week posted** | **Time posted** |
| 1 | Involving public contributors from the very start | Co-producing | #ICTMC2022 we asked @MRCNIHRTMPR students to complete this sentence on a leaf “how we can best engage patients the public with trials methodology?" and the branches flourished. One idea was an advent calendar so here it is! Day 1 involving public contributors from the start. Share your ideas or activities too | 25 | 6 | 57 | 1 | 7143 | 218 | "By understanding the value and thinking it's the right thing to do" | Thursday | 17.57 |
| 2 | Advertising trials methodology to the general public including how they can get involved (including billboards, social media, radio etc) | Informing/ inspiring | Advent calendar day 2 - What advertising have you used for trials methodology or, if you are a patient/member of the public what advertising do you think works best? #trialsmethodology #clinicaltrials | 11 | 1 | 13 | 1 | 1649 | 108 |  | Friday | 9.28 |
| 3 | Creating content that will engage the public and sharing it where people are most likely to access it | Informing /inspiring | Advent calendar day 3 - Do you create content for trials methodology? What type of content do you make and where do you share it? What type of content do you engage with personally? #trialsmethodology #clinicaltrials | 11 | 0 | 12 | 0 | 1279 | 64 | "Think about the benefits, risks and concerns that exist and start the conversation around those as these provide purpose to the initial dialogue" | Saturday | 10.16 |
| 4 | Engage with children at school to talk about trials and trials methodology | Stimulating thinking | Advent calendar day 4 - We know @HRBTMRN have done a great job of this. Have you done any school outreach? What have you found works well? #trialsmethodology #clinicaltrials | 14 | 1 | 23 | 0 | 1911 | 89 |  | Sunday | 9.56 |
| 5 | Engaging the public on general topics like access to their health record and methods work around this - relevant to majority of people and not condition specific | Stimulating thinking | Advent calendar day 5 - Access to electronic health records is something that affects lots of people. How could we use this topic to start a bigger conversation about trials and trials methodology? #trialsmethodology #clinicaltrials | 12 | 0 | 12 | 0 | 1260 | 62 | "and how do they feel about these records as opposed to all the other data on them, from ONS to mobile phones and retail reward cards" | Monday | 10.33 |
| 6 | Engaging with communities and community leaders | Collaborating | Advent calendar day 6 - Talking to different communities and community leaders to share information and listen to information needs. Do you have any top tips to share? #trialsmethodology #clinicaltrials | 14 | 0 | 18 | 0 | 1382 | 83 |  | Tuesday | 10.39 |
| 7 | Having a clear and transparent process for trials and trials methodology | Informing/  inspiring | Advent calendar day 7 - Having a clear and transparent process about trials and trials methodology. What do we need to do to achieve this? What do people want to know? #trialsmethodology #clinicaltrials | 10 | 0 | 9 | 0 | 780 | 58 |  | Wednesday | 9.29 |
| 8 | Having a general forum for people to ask questions and share experiences | Informing/  inspiring | Advent calendar day 8 - Having a forum to ask questions and share ideas. What would this look like? Who would the moderators be? #trialsmethodology #clinicaltrials | 6 | 0 | 5 | 0 | 711 | 25 |  | Thursday | 11.07 |
| 9 | Having endorsement from celebrities/public figures/patient organisations | Informing/  inspiring | Advent calendar day 9 - Having endorsement from celebrities/public figures/patient organisations? Are you one of these and would like to talk more about how you can help engage patients and the public about trials methodology? We'd love to hear from you! #trialsmethodology #clinicaltrials | 7 | 0 | 5 | 0 | 833 | 33 |  | Friday | 12.26 |
| 10 | Having public contributor champions who will share their experience and encourage involvement | Informing/  inspiring | Advent calendar day 10 - Having public contributor champions who will share their experience and encourage involvement. There are some amazing trials methodology champions (tag). What support does this role need? #trialsmethodology #clinicaltrials | 8 | 0 | 7 | 0 | 1029 | 43 |  | Saturday | 13.26 |
| 11 | Increasing accessibility including language, cultural considerations, use of plain language | Co-producing  Collaborating | Advent calendar day 11- Increasing accessibility including language, cultural considerations, use of plain language. What does accessibility look like? What changes have you made or what are your top tips for accessible information? #trialsmethodology #clinicaltrials | 10 | 0 | 14 | 0 | 1206 | 65 |  | Sunday | 20.01 |
| 12 | Linking to current issues around climate change and how trials are thinking about their impact | Stimulating thinking | Advent calendar day 12 - Linking to current issues around climate change and how trials are thinking about their impact. #ICTMC22 included really interesting talks about the carbon footprint of trials. Could this be a way to engage the general public in methodology? #trialsmethodology #clinicaltrials | 11 | 0 | 16 | 0 | 1164 | 53 |  | Monday | 13.22 |
| 13 | Offering fair payment for public contributors | Co-producing  Collaborating, Informing decision making | Advent calendar day 13 - Offering fair payment to public contributors. What does fair payment look like? What other support is needed i.e. childcare, carers, accessible meetings? #trialsmethodology #clinicaltrials | 12 | 1 | 12 | 1 | 1557 | 63 |  | Tuesday | 18.03 |
| 14 | Promoting the impact of trials on individuals and the public and why methods are important | Stimulating thinking  Informing/  inspiring | Advent calendar day 14 - Promoting the impact of trials on individuals and the public. What are the benefits of well designed trials? How does trials methodology research contribute to this? #trialsmethodology #clinicaltrials | 10 | 0 | 10 | 0 | 1195 | 47 | "improving the research methods makes all research studies more efficient" | Wednesday | 18.03 |
| 15 | Sharing the experiences of patients and the public already involved in research including who has been involved | Stimulating thinking  Informing/  inspiring | Advent calendar day 15 -Sharing the experiences of people involved in research. What has it been like to take part? What impact has ion those involved? Who has been involved? #trialsmethodology #clinicaltrials | 7 | 0 | 10 | 1 | 886 | 37 |  | Thursday | 21.13 |
| 16 | Sharing best practice and methods with other researchers | Informing/  inspiring | Advent calendar day 16 - As Trials Methodologists how can we share ideas and resources with one another? A Core Outcome Set PPI Toolkit will be launching early in 2023 as part of the @CometInitative Are there any other toolkits for trials methodology? | 12 | 0 | 14 | 0 | 1594 | 68 |  | Friday | 11.35 |
| 17 | Sharing information about trials generally with the general public | Stimulating thinking  Informing/  inspiring | Advent calendar day 17 - Some of the earlier entries relate to this, sharing information with the pubic generally and not just those who are invited to take part in research. Have you used TikTok to share information about trials? | 5 | 0 | 11 | 0 | 1027 | 38 |  | Saturday | 19.2 |
| 18 | Sharing skills public contributors have and why they are so important in trials | Stimulating thinking  Informing/  inspiring | Advent Calendar day 18 - Public contributors have a range of skills that are of huge value to research and sharing these skills and how they interact with the role in trials methodology is important | 8 | 0 | 8 | 0 | 1231 | 37 |  | Sunday | 14.55 |
| 19 | Sharing the results of studies to a wide audience | Co-producing  Collaborating  Stimulating thinking  Informing/  inspiring | Advent calendar Day 19 – Sharing the results of studies with a wide audience, broader than participants and patients and perhaps the public more generally? Where would you share this? | 8 | 0 | 5 | 0 | 1156 | 43 |  | Monday | 12.2 |
| 20 | Understanding public contributor needs and how they can be met | Co-producing  Collaborating  Informing decision making | Advent calendar day 20 – How can we better support someone in a PPI role? | 10 | 0 | 15 | 0 | 1659 | 57 |  | Tuesday | 17.37 |
| 21 | Using art and creative/fun methods to engage the general public | Stimulating thinking  Informing/  inspiring | Advent calendar day 21- What can we learn from creative practice and how can we apply it to engagement activities | 6 | 1 | 5 | 0 | 1175 | 31 |  | Wednesday | 20.39 |
| 22 | Using tv drama or documentaries to engage the general public | Informing/  inspiring | Advent calendar day 22 - Would you watch a drama or documentary about clinical trials? Who would star in it or present it? | 7 | 0 | 9 | 0 | 1666 | 35 |  | Thursday | 22.33 |
| 23 | Sharing the history of trials/trials methods research and the impact on society | Informing/  inspiring | Advent calendar day 23 - The success stories of trials and methods can be taken for granted, so we need to ensure we share them. Do you know the story of the Cochrane logo? An image with a great message about the impact of evidence synthesis | 7 | 0 | 5 | 0 | 1507 | 27 |  | Friday | 9.2 |
| 24 | Continuing to work together as a community to support patient and public involvement and engagement | Informing/  inspiring  Consulting  Collaborating  Stimulating thinking | Advent calendar day 24 - It’s the last day so for this post we want to with you happy holidays! We look forward to seeing some of these ideas become reality in 2023 | 6 | 0 | 5 | 0 | 1305 | 27 |  | Saturday | 14.28 |
